# Illinois Doctor

**Published:** 1872-10

**Authors:** 


A DOCTOR recently settled in Illinois, and the first case he had was a boy, who, while shelling pop-corn, got a kernel in his windpipe. The doctor examined the case carefully, looked at the patient's tongue, and then told the father of the boy to build up a hot fire. When that was done the doctor told them to take the boy and hold him over the fire until the kernel got hot enough to "pop out." The old man went up-stairs and got his shot-gun, but while he was loading it the doctor escaped.

